# Solidification and Biotoxicity Assessment of Thermally Treated Municipal Solid Waste Incineration (MSWI) Fly Ash

**DOI:** 10.3390/ijerph14060626

**Published:** 2017-06-10

**Authors:** Bing Gong, Yi Deng, Yuanyi Yang, Swee Ngin Tan, Qianni Liu, Weizhong Yang

**Affiliations:** 1 School of Materials Science and Engineering, School of Chemical Engineering, Sichuan University, Chengdu 610065, China; borg_gongbing@163.com (B.G.); dengyibandeng@scu.edu.cn (Y.D.); qiannilu@163.com (Q.L.); 2 Department of Materials Engineering, Sichuan College of Architectural Technology, Deyang 618000, China; boyyangyuanyi@126.com; 3 National Sciences and Science Education, National Institute of Education, Singapore 637616, Singapore; sweengin.tan@nie.edu.sg

**Keywords:** MSWI fly ash, thermal treatment, toxicity characteristic leaching procedure (TCLP), microbial assay

## Abstract

In the present work, thermal treatment was used to stabilize municipal solid waste incineration (MSWI) fly ash, which was considered hazardous waste. Toxicity characteristic leaching procedure (TCLP) results indicated that, after the thermal process, the leaching concentrations of Pb, Cu, and Zn decreased from 8.08 to 0.16 mg/L, 0.12 to 0.017 mg/L and 0.39 to 0.1 mg/L, respectively, which well met the limits in GB5085.3-2007 and GB16689-2008. Thermal treatment showed a negative effect on the leachability of Cr with concentrations increasing from 0.1 to 1.28 mg/L; nevertheless, it was still under the limitations. XRD analysis suggested that, after thermal treatments, CaO was newly generated. CaO was a main contribution to higher Cr leaching concentrations owing to the formation of Cr (VI)—compounds such as CaCrO_4_. SEM/EDS tests revealed that particle adhesion, agglomeration, and grain growth happened during the thermal process and thus diminished the leachability of Pb, Cu, and Zn, but these processes had no significant influence on the leaching of Cr. A microbial assay demonstrated that all thermally treated samples yet possessed strong bactericidal activity according to optical density (OD) test results. Among all samples, the OD value of raw fly ash (RFA) was lowest followed by FA700-10, FA900-10, and FA1100-10 in an increasing order, which indicated that the sequence of the biotoxicity for these samples was RFA > FA700-10 > FA900-10 > FA1100-10. This preliminary study indicated that, apart from TCLP criteria, the biotoxicity assessment was indispensable for evaluating the effect of thermal treatment for MSWI fly ash.

## 1. Introduction

Due to the marked rise in economy and population, billions of tons of municipal solid waste (MSW) are generated annually worldwide, and the amount is soaring [1]. Traditional disposal of MSW in landfills has induced severe deterioration of quality of air, underground water, public health, etc. [2]. Therefore, municipal solid waste incineration (MSWI) is considered the most effective alternative to the landfill disposal of MSW. Generally, MSWI can considerably reduce the volume and weight of solid waste by 90% and 70%, respectively, and realize energy recovery and disinfection [3]. However, the generation of fly ash during the combustion process is another big nuisance, which poses a grave menace to environmental quality. Moreover, on account of the high concentrations of trace metals, fly ash is classified as hazardous wastes, and thus effective detoxification is indispensable before its reuse or disposal in landfills.

Thermal treatment is widely adopted to suppress the leachability of heavy metals in MSWI fly ash [4]. Thermal treatment technologies are commonly divided into three types: vitrification, melting/fusing, and sintering [5]. Vitrification melts fly ash with glass and/or other wastes to form homogeneous liquid phase, and then to obtain amorphous, single-phase glass. The objective of melting/fusing processes is to get heterogeneous products consisting of glassy materials and crystalline phases. The objective of the sintering process is to densify the porous solid particles below the melting points of their main components. To determine whether the treated slags are hazardous wastes or not, the extensively recognized assessment method—the toxicity characteristic leaching procedure (TCLP) leaching test—is implemented in many cases. For example, Wey et al. [6] investigated the continuous sintering behavior of fly ash with a rotary kiln. According to TCLP results, the leaching concentrations of heavy metals in leachate from residue sintered at 900 °C were below the regulations in Taiwan. Similar leaching results were reported in many previous studies [7,8,9]. However, the biotoxicity of thermal products derived MSWI fly ash has been seldom considered and reported. Undoubtedly, the evaluation of biotoxicity of thermally treated residues is a crucial item. A few papers, for example, have suggested that, though the TCLP leaching concentrations of heavy metals from cyclone ash, scrubber ash, and bottom ash met relevant regulations, their leachates still showed evident biotoxicity toward experimental subjects such as *Escherichia coli* DH5, *Bacillus subtilis*, wheat seeds, etc. [10,11,12,13]. Furthermore, fly ash would suffer a high-temperature thermal process when it was reutilized as an alternative to some mineral materials for producing construction materials such as ceramic bricks. These products had a direct contact with environment or even human bodies. As a consequence, the products containing MSWI fly ash should not only meet the TCLP criteria but also have a good biocompatibility.

In this research, MSWI fly ash from the baghouse of a waste-to-energy plant was sintered under different temperatures. In addition, conventional leaching procedure (HJ/T 300-2007 [14]; for comparison purpose) and an in vitro bacterial inhibition test against *Escherichia coli* (*E. coli*, ATCC 25922) and *Staphylococcus aureus* (*S. aureus*, ATCC 25923) were conducted to evaluate the chemical stability of different thermal products. In this novel method, direct contact between bacteria and thermally treated residues was involved, which apparently showed the effect of thermally treated wastes on the growth of two kinds of bacteria.

## 2. Materials and Methods

MSWI fly ash samples were collected from a waste-to-energy plant with an incineration capacity of 1800 tons per day located in Chengdu, China. The air pollution control (APC) system of the incinerator was a semi-dry lime scrubbing device equipped with a fabric filter. The raw APC residue was first sampled from the fly ash silo of the MSWI plant. Then, the collected samples were dried to constant weight at 105 ± 0.5 °C for 24 h and sealed in a polyethylene drum for subsequent experiments. All starting materials derived from the same batch.

The pure origin fly ash was directly pressed at 20 MPa in a cylindrical steel die with a dwell time of 1 min to obtain discs of 8 mm in diameter and 2 mm in height. The discs were sintered in air under different temperatures varying from 700 to 1100 °C with an interval of 200 °C, a heating rate of 5 °C/min and a holding time of 10 min followed by natural cooling. These samples were denoted as follows: raw fly ash (RFA), FA700-10 (fly ash treated at 700 °C for 10 min), FA900-10 (fly ash treated at 900 °C for 10 min), and FA1100-10 (fly ash treated at 1100 °C for 10 min), respectively.

The TCLP leaching test, following China EPA method HJ/T 300-2007, was employed to assess the leaching properties of trace metals in nascent fly ash and thermally treated residues. The thermally treated discs were ground to pass through a 1 mm standard sieve. Because the pH values of the leachate exceeded 5.0, the extractant with a pH of 2.64 ± 0.05 was chosen for the leaching test. The extraction solution was prepared by diluting 17.25 mL of glacial acetic acid with 1 L of deionized water. In the leaching procedure, 5 g of solid samples were well mixed with an extraction solution in a liquid-to-solid ratio of 20 in a 2 L polyethylene bottle, and the slurry was then agitated at a rotation rate of 30 rpm for 18 h. The resulting solution was filtered through a 0.45 μm membrane disk, and the filtered solution was acidified with nitric acid (1 M) up to pH = 2. Ten milliliters of final extract was injected into the polyethylene tube, and preserved at 4 °C prior to chemical analysis.

Microbial assay was performed to study biotoxicity of thermally treated fly ash. Before microbial assay, all utensils and samples were sterilized via moist–heat method (121 °C at 15 psi for 20 min) to exclude the presence of unwanted bacterial contaminants. Sterile LB broth and LB agar plates were prepared using deionized water according to the standard procedure [15]. In the microbial test, LB agar plates seeded with bacteria were used to measure the zones of inhibition. Firstly, the bacteria suspension was diluted with LB broth to 10^7^ CFU/mL. Then, the diluted bacteria suspension was smeared onto LB agar plates with sample discs laid on the surface of LB agar plates. Afterwards, LB agar plates associated with the samples and bacteria suspension were incubated in an oven at 37 °C for 24 h. In addition, to assess the effect of treated or untreated discs on the growth of bacteria as time increasing, the discs were immersed in LB broth seeded with bacteria, and the mixture was incubated in a 48-well cell culture plate at 37 °C. One hundred microliters of culture medium from each well was collected after 0, 3, 6, 9, and 12 h of incubation for the optical density (OD) test at 600 nm. OD values were determined using a microplate reader (SAF-680T, BAIIU, Shanghai, China). OD values can reflect concentrations of bacteria in aqueous solution, and higher concentrations of bacteria implied higher OD values. Therefore, when bacteria were able to grow in the medium, the concentration of bacteria in the medium ascended, resulting in the enhancement of OD values. To the contrary, when bacterial growth was inhibited, the OD values decreased. 

The particle size distribution analysis of RFA was conducted using a laser diffraction particle size analyzer (LPSA; JL-1177, Jingxin Powder Test Equipment Co., Ltd., Chengdu, China) with deionized water as dispersing media. The concentration was approximately 0.1 mg solid/mL water. The raw fly ash was mixed with deionized water in a glass beaker, and the suspension was then subject to ultrasonic dispersion before the test. An X-ray fluorescence spectrometry (XRF; Shimadzu sequential XRF-1800, Shimadzu, Japan) was used to determine main chemical components in RFA. The concentrations of trace metals in fly ash were detected by inductively coupled plasma-atomic emission spectroscopy (ICP-AES; IRIS Advantage, Thermo 22 Jarrell Aha Corporation, Boston, USA) after the fly ash was digested according to US EPA method 3050B [16]. Similarly, leaching concentrations of Pb, Cu, Cr, and Zn from residues were also tested by the ICP-AES. Principal crystalline phase analysis was implemented by X-ray powder diffraction (XRD; LabX XRD-6100, Shimadzu, Japan) with Cu Kα radiation (λ = 0.15418 nm) and scanning ranging from 10 to 80° at a scan rate of 1°/min. A scanning electron microscope (SEM; JSM-5900LV, JEOL, Tokyo, Japan) was employed to observe morphology characteristics. Samples were coated with gold for 30 s before SEM observation.

## 3. Results and Discussion

### 3.1. Characterization of Raw Fly Ash

**Figure 1:**
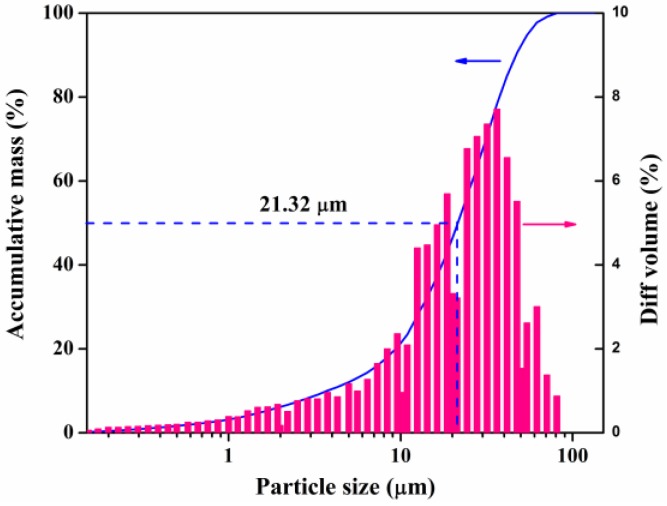
Particle size distribution of raw fly ash.

Particle size distribution of RFA was presented in Figure 1. The average D(50) particle size (50% of the particle sizes are smaller than this value) was 21.32 μm. In order to analyze main chemical components of raw MSWI fly ash, XRF analysis was performed, and the results are presented in Table 1. It is obvious that calcium oxide (CaO) and Cl were the two most dominant compositions, accounting for 53% and 20%, respectively. In the APC system, a huge amount of lime slurry was injected to absorb flue gas, which was the most important resource of CaO. As for Cl, during the incineration of MSW, metal chlorides, and hydrogen chloride (HCl) were generated and got into flue gas, which accounted for the high content of Cl in fly ash. The total concentrations of several selected trace metals associated with fly ash samples including Pb, Cr, Cu, and Zn were 159.9 mg/kg, 106 mg/kg, 383.2 mg/kg, 365.1 mg/kg, respectively. Comparable elemental concentrations of heavy metals in MSWI fly ash have been reported in other literature [17,18].

### 3.2. Leaching Concentrations of Heavy Metals

In the present study, temperature was found to have a significant influence on leaching behavior of heavy metals in fly ash. Table 2 shows leaching concentrations of heavy metals and corresponding limits in the identification standard for hazardous wastes (S1, GB 5085.3-2007) and the pollution control standard for sanitary landfill (S2, GB16889-2008). Evidently, Pb in RFA was detected to have the highest leaching concentration of 8.08 mg/L followed by Zn, Cu, and Cr. The leaching concentration of Pb in RFA far exceeded the limits in S1 and S2, which indicated that the fly ash ranked within hazardous waste and could not be directly disposed in landfill. However, the leaching concentrations of Cr, Cu, and Zn in RFA well met two kinds of standards. After thermal treatment, leaching concentrations of Pb, Cu, and Zn were significantly reduced. As temperature increased from 700 to 900 °C, leaching concentrations of Pb, Cu, and Zn were diminished from 8.08 to 0.16 mg/L, 0.12 to 0.017 mg/L, and 0.39 to 0.1 mg/L, respectively. It was interesting that leaching concentration of Cr increased from 0.1 to 1.28 mg/L when temperature ranged from 700 to 900 °C, but was still under the regulations of Cr. Furthermore, leaching behavior of Pb, Cu, and Zn was further suppressed as temperature increased, which was different from that of Cr. These results are highly consistent with other literature [7,19].

### 3.3. Mineralogy of Raw and Thermally Treated Fly Ash

**Figure 2:**
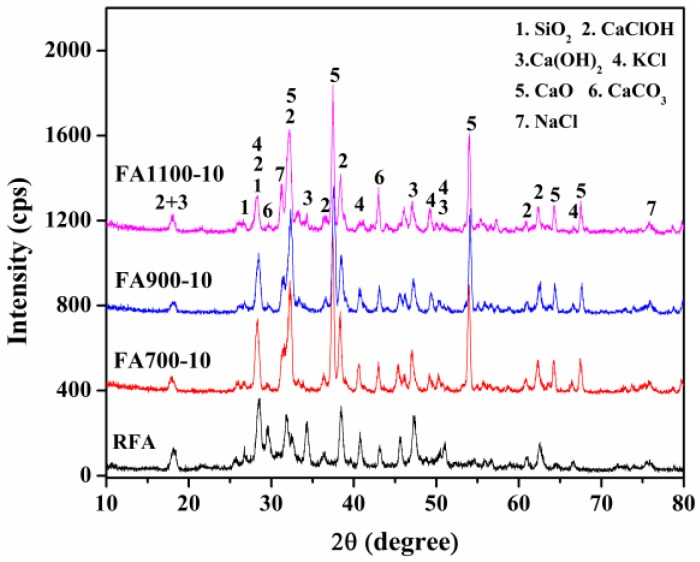
XRD spectra of raw fly ash (RFA) and thermally treated slags.

Figure 2 depicted XRD patterns of RFA and thermal products under different conditions. The XRD spectrum suggested that the main mineral compositions, identified in RFA, were Ca(OH)_2_, SiO_2_, NaCl, etc. the result, to some degree, was in agreement with Table 1. As shown in Figure 2, except mineral phases in RFA, the peaks of CaO were detected in FA700-10, FA900-10, and FA1100-10. During thermal treatment, Ca(OH)_2_, CaClOH, and CaCO_3_ were decomposed coupled with the appearance of CaO in thermally treated slags and the release of H_2_O and/or CO_2_. Ca compounds were transformed into the aluminosilicates with a network structure, which were conducive to suppressing the leaching of heavy metals in treated wastes. However, the superfluous generation of CaO enhanced the leaching behavior of Cr. Because CaCrO_4_ was newly formed, and was more liable to leach compared to Cr(Ш)-compounds [20,21]. By comparing the XRD patterns of RFA and heated wastes, it was observed that crystallization in heated samples presumably was strengthened. The insight was approved by the overall increase in peak intensities of crystalline phases and the slightly narrow effect of the spectrum between the 2-theta of 20~40° compared with the nascent fly ash. Better crystallization, hence, was also beneficial for reducing leaching concentrations of heavy metals through confining the heavy metals in the lattice and grain boundary.

### 3.4. Morphology of Raw and Thermally Treated Fly Ash

**Figure 3:**
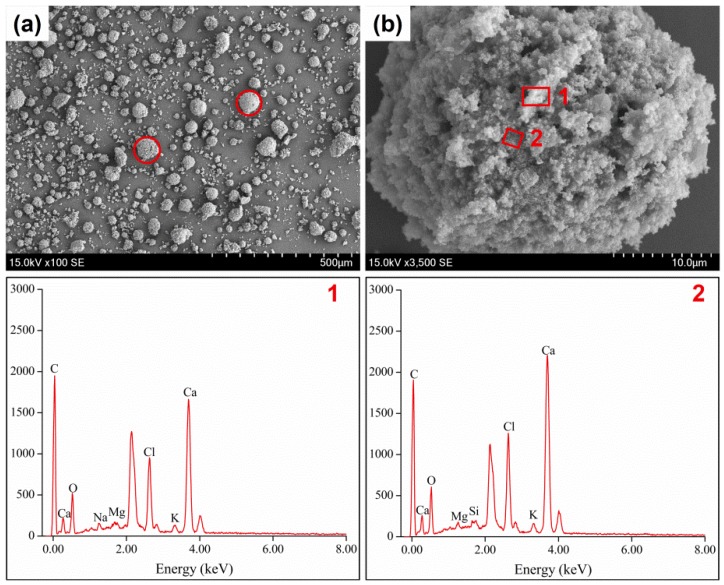
SEM and EDS images of RFA: (**a**) low-magnification image; (**b**) high-magnification image.

**Figure 4:**
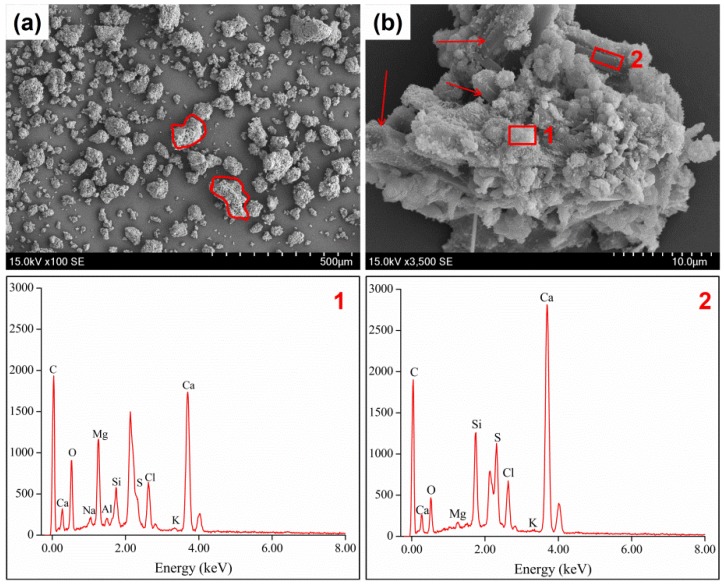
SEM and EDS images of FA900-10: (**a**) low-magnification image; (**b**) high-magnification image.

Figure 3 showed morphologies and components characteristics of RFA. From Figure 3a, we could observed that most RFA particles had spherical shapes that contributed to a larger leaching area and made the heavy metals prone to leach. As shown in Figure 3b RFA particles contained many granular crystallites accompanied with large quantities of pores. Obviously, these granular crystallites and pores resulted in high leaching concentrations of heavy metals. Furthermore, EDS images demonstrated that compositions of different area of RFA particles were similar. Figure 4 revealed SEM images and corresponding EDS results of the heated sample (FA900-10). Similarity in morphology can be detected among FA900-10, FA700-10, and FA1100-10. Comparatively, great changes in fly ash morphology took place after thermal treatment. As highlighted in Figure 4a, after thermal treatment, anomalous particles came into being due to particle adhesion and conglomeration. These processes encapsulated heavy metals in newly generated irregular particles and thus restrained the leaching of heavy metals [6]. In addition, Figure 4b showed that columnar grains in a large size were formed in heated samples. During the heating process, the grains grew to a greater size in comparison to that of RFA. In the growing procedure, parts of the heavy metals were transformed into a crystal lattice, which led to decreases in their leachability. Remarkably, it could also be noticed from Figure 4b that some small crystallites conglomerated onto the surface of big columnar grains reducing leaching concentrations of heavy metals. EDS results of the thermal product suggested that crystallites with different shapes varied in components. In particular, big columnar grains were abundant in C, Ca, O, Mg, Si, S, and Cl, while small crystallites, except for the aforementioned elements, also contained Na and Al.

### 3.5. Biotoxicity Assessment

**Figure 5:**
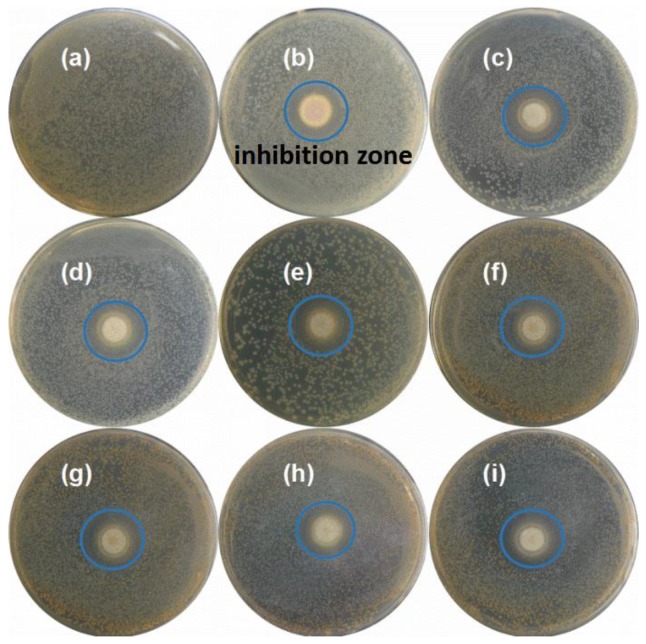
Bacterial inhibition test for RFA and thermally treated disks against *E. coli* and *S. aureus*. (**a**) The growth of *E. coli* without fly ash disk; (**b**–**e**) the growth of *E. coli* in the presence of RFA, FA700-10, FA900-10, and FA1100-10, respectively; (**f**–**i**) the growth of *S. aureus* in the presence of RFA, FA700-10, FA900-10, and FA1100-10, respectively.

**Figure 6:**
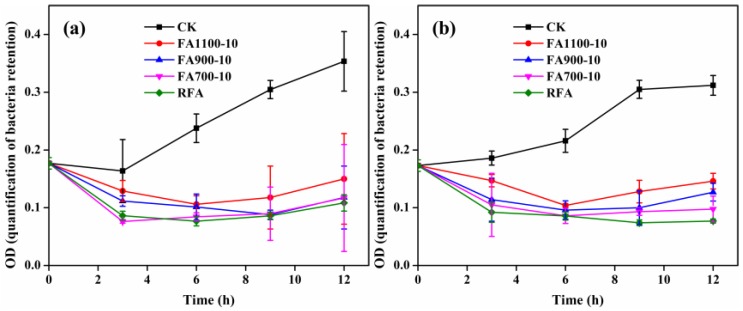
Time-killing curves of RFA, FA700-10, FA900-10, and FA1100-10 against (**a**) *E. coli* and (**b**) *S. aureus.*

According to Table 2, it could be concluded that Pb, Cu, and Zn became less leachable after the thermal process, whereas the leaching concentration of Cr increased, yet it was still under the limitations. These results proved that the treated slags were not hazardous waste and even could be disposed in landfills. However, the biocompatibility of the thermally treated slags was not satisfactory. To study the biotoxicity of thermally treated residues, two kinds of bacteria were incubated in the presence of fly ash plates treated under different conditions, and the results were exhibited in Figure 5. We could see that *E. coli* colonies relatively evenly distributed in the plate without fly ash disk (Figure 5a), which was the same with *S. aureus* (the data was not displayed here). However, clear bacterial inhibition zones could be observed in Figure 5b–i, which highlighted that all samples, treated or untreated, at different temperatures harbored strong antibacterial activity against *E. coli* and *S. aureus*. Figure 6 presents the time-killing curves of RFA, FA700-10, FA900-10, and FA1100-10 against *E. coli* (Figure 6a) and *S. aureus* (Figure 6b), which describes the bactericidal ability of all samples as time increases. Bacterial growth could be reflected by determining the optical density (OD) of the culture media at 600 nm. Obviously, in the absence of fly ash disk (CK curves), OD values of media containing *E. coli* or *S. aureus* increased to about 0.35 after 12 h. As shown in Figure 6, however, FA1100-10 showed bacterial inhibition toward both *E. coli* and *S. aureus* within 6 h, while this inhibition was released after 6 h with a slight increase in OD values. Similarly, FA900-10, FA700-10, and RFA restrained the growth of two bacteria after a certain period of incubation, and then failed to inhibit bacterial regrowth. It was worth noting that the regrowth, after 12 h, reached a lower level in the presence of FA900-10, FA700-10, and RFA compared with FA1100-10, which demonstrated that FA1100-10 showed relatively lower biotoxicity. Generally, the release of poisonous species from the incineration ash into the environment posed a great threat to organism as revealed by Ribe et al. [22]. In the present work, *E. coli* and *S. aureus* were cultured in the presence of treated or untreated fly ash disks, respectively. In this process, heavy metal ions were released from the fly ash disks. These heavy metals worked on enzymes in bacterial cells and caused it to lose activity, which inhibited the growth of bacteria. Higher concentrations of heavy metals, which were related to leaching capacity, implied stronger inhibition. In addition, exposure to heavy metals induced great DNA damage and mutagenesis [13,23]. Moreover, the pH of leachate from all samples reached a significantly high level of about 12.5, which also contributed to bactericidal activity through changing osmotic pressure of cell cytomembrane.

Much literature had focused on the biosecurity of RFA [13,24,25,26]. These studies incubated organisms, such as bacteria, cells, and plants, in the presence of leachate from RFA, which could be classified as an indirect method. Indeed, aforementioned research provides references for the biosafety of pristine fly ash. However, direct contact between organisms and thermal products (such as concrete and ceramic) derived from fly ash was inevitable, so biotoxicity assessment of thermally treated fly ash was indispensable. 

## 4. Conclusions

In the present work, a thermal procedure was employed for fly ash detoxification using TCLP leaching test and bacteria culture assay as evaluating methods. After thermal treatment, leaching concentration of Pb significantly decreased to below the regulations and that of Cu and Zn also showed a downward trend. However, after thermal treatment, Cr became more leachable, but the leaching concentration of Cr was still acceptable through TCLP standards. Contradictorily, the results of the bacteria inhibition assay indicated that all samples, including RFA and thermally treated fly ash disks, showed obvious inhibition toward the growth of both *E. coli* and *S. aureus*. Especially, FA1100-10 showed the lowest biotoxicity against *E. coli*, while biotoxicities toward *E. coli* of RFA, FA700-10, and FA900-10 were close to each other. In addition, RFA inflicted the greatest inhibition on the growth of *S. aureus* followed by FA700-10, FA900-10, and FA1100-10 in a descending sequence.

This work provided a preliminary idea about the necessity of evaluating whether thermal products from fly ash, which well met TCLP criteria, was biocompatible. Undoubtedly, much additional work is needed to optimize thermal treatment technologies such that thermal products derived from MSWI fly ash are environmentally friendly. Moreover, a special evaluation system should be established to assess the biocompatibility of products derived from MSWI fly ash.

## Figures and Tables

**Table 1:** Basic compositions of raw fly ash.

Composition	CaO	NaO	SO_3_	SiO_2_	MgO	Fe_2_O_3_	Al_2_O_3_	TiO_2_	P_2_O_5_	Cl
Ratio (%)	53.0	7.7	5.9	3.9	3.8	1.9	1.0	0.5	0.3	19.9

**Table 2:** Leaching concentrations of the selected heavy metals and corresponding limits (mg/L).

Samples	Leaching Concentrations (mg/L)			
	Pb	Cr	Cu	Zn
Raw Fly Ash (RFA)	8.08	0.10	0.12	0.39
FA700-10	1.12	0.73	0.04	0.17
FA900-10	0.30	0.70	0.013	0.09
FA1100-10	0.16	1.28	0.017	0.10
Limits in S1	5	5	100	100
Limits in S2	0.25	1.5	40	100
